# Incidental vocabulary learning from digital games: the predictive role of game playing time, foreign language motivation, and anxiety

**DOI:** 10.3389/fpsyg.2026.1798613

**Published:** 2026-05-14

**Authors:** Serdar Akbulut, Bilal Şimşek, Bekir Direkci, Betül Koparan, Mevlüt Gülmez, Emine Ela Şimşek, Gökhan Kayır

**Affiliations:** 1 Department of Turkish Language Education, Faculty of Education, Manisa Celal Bayar University, Manisa, Türkiye; 2 Department of Turkish Language Education, Faculty of Education, Akdeniz University, Antalya, Türkiye; 3 Department of Early Childhood Education, Faculty of Education, Akdeniz University, Antalya, Türkiye; 4 Department of Measurement and Evaluation, Faculty of Education, Manisa Celal Bayar University, Manisa, Türkiye

**Keywords:** anxiety, digital games, incidental vocabulary learning, L2 motivation, language learning

## Abstract

**Introduction:**

This study examined the effect of digital games on incidental second language (L2) vocabulary learning and identified the factors predicting incidental word learning through digital gameplay.

**Methods:**

Data were collected in two stages. In the first stage, 1,204 middle school students participated, including 603 students who played digital games and 601 who did not. An achievement test consisting of vocabulary items that could be incidentally learned from a digital game was administered. In the second stage, analyses focused only on the 603 students who played digital games. Their vocabulary knowledge, L2 learning motivation, anxiety levels, and digital game playing duration were examined.

**Results:**

The results indicated that students who played digital games performed significantly better than those who did not. Correlation analyses revealed a moderate positive relationship between vocabulary test scores and gaming duration, as well as a positive relationship between vocabulary knowledge and motivation. In contrast, vocabulary test scores were negatively correlated with anxiety. Hierarchical regression analysis showed that digital game playing time was the strongest predictor of incidental vocabulary learning. In the final model, which included gaming duration, motivation, and anxiety, the explained variance reached 29.8%.

**Discussion:**

Overall, the findings suggest that digital games may be associated with incidental L2 vocabulary learning and may provide environments where such learning can occur. In addition to exposure frequency, affective factors such as motivation and anxiety play a significant role in this process. Therefore, the study underscores the importance of incorporating psychological variables when designing and implementing digital game-based language learning environments.

## 1 Introduction

With today's technological advances, tools have become integrated into every aspect of our lives; innovations have taken place in many areas, such as health, transport and education. Once developed and widely adopted in societies, technological tools can ultimately be used in a variety of fields. One of the most obvious examples of this is digital games. Originally released for entertainment purposes, these games were later considered worthy of study and research in many disciplines. One of the main reasons for this is that digital games appeal to a large global audience and have a large player base in different regions and age groups. Research in the five largest European countries (the United Kingdom, Germany, France, Spain and Italy) shows that around 50% of people aged 6 to 64 play digital games (Interactive Software Federation of Europe—ISFE, 2021). It is noteworthy that a significant proportion of 18- to 24-year-olds use these games for leisure and stress relief (China News Network, 2022), while the average weekly playing time for 11- to 14-year-olds is around 9.5 h. The global gaming industry is rapidly developing (Li et al., 2025), one in three people identify themselves as a “gamer” (Ural, 2009), and the number of gamers is approaching 3 billion (Dimitrievksi, 2025). In this context, digital games have gone beyond mere entertainment products and have come to be considered highly interactive spaces (Hardof-Jaffe and Amzalag, 2025) and learning environments offering rich, context-oriented language input (Wang et al., 2025).

Digital games have an extensive scope and encompass many features depending on their context. Many different digital game concepts can be developed, such as problem-solving-based, interaction-based, and task-oriented. Research has shown that these games can support learning in fields such as health (McHale et al., 2012), mathematics (Kebritchi et al., 2010), STEM (Boyle et al., 2016), and social sciences. In this context, digital games in the literature are generally categorized into two main groups: educational or serious games and commercial entertainment games (Breuer and Bente, 2010; Schrader, 2023). While educational games are designed based on pedagogical principles in line with specific learning objectives, commercial games primarily aim to provide entertainment and enhance user experience and therefore do not include direct instructional goals. Nevertheless, commercial games can also offer important opportunities for incidental language learning through rich contextual language input, repeated exposure to vocabulary, and interaction among players. This study specifically focuses on the incidental vocabulary learning process that emerges within the context of commercial digital games. Digital games may be designed specifically for educational purposes, or commercial entertainment games may incidentally offer learning opportunities. In both contexts, games that provide context-rich language input and support written as well as oral interaction are regarded as promising tools for language learning (Li et al., 2021). However, the role of digital games in language learning should be examined from a multidimensional perspective. Mere exposure to a wide range of lexical items within a game-based environment does not, by itself, ensure effective vocabulary acquisition. Nevertheless, prior research consistently demonstrates that digital games can enhance learners' motivation and reduce language learning anxiety (Connolly et al., 2011; Yang et al., 2024). In light of these findings, digital games appear to positively influence individuals' affective processes in language learning. As language learning unfolds over an extended period, sustained learner involvement becomes essential. From this perspective, digital games foster participation and interaction in the learning process (Lee, 2019) and demonstrate potential for supporting the development of language skills and vocabulary (Jia et al., 2024; Kazu and Kuvvetli, 2023).

Vocabulary is critically important in the language-learning process, especially for second-language learners, who use words as the fundamental building blocks of language (Ghazal, 2007). Without sufficient vocabulary, the underlying concepts of given words cannot be understood, rendering syntax and discourse knowledge almost useless (Schmitt et al., 2021). Vocabulary develops in three dimensions: form, meaning, and usage (Nation, 2001). This process occurs both formally and systematically in schools and, incidentally, through exposure in social life. Incidental vocabulary learning can occur in many areas of social life. Research shows that individuals can also learn vocabulary incidentally when exposed to an engaging virtual environment (Lai and Chen, 2023). However, incidental vocabulary learning is not merely a process that can be explained by the frequency of exposure to input. This type of learning occurs through various cognitive mechanisms related to how the learner processes the language input. First and foremost, it is considered a fundamental condition for learning that the learner notices the new words present in the language input and directs their attention to these elements (Leow, 2019; Schmidt, 1990; Winke, 2013). The noticed words are then contextualized, and learners create mental associations between form and meaning, while establishing connections between their existing mental lexicons and new inputs (Segal and Dobson, 1992). Furthermore, repeated encounters in meaningful contexts contribute to strengthening the mental representations of words and making them more permanent in long-term memory (Bisson et al., 2014; Rott, 1999; Sahin and Yavuz, 2025; Webb et al., 2023; Zhang, 2022). Particularly in interactive environments such as digital games, task-oriented attention, problem-solving processes, and high levels of motivation enable learners to process the language input at a deeper level, thereby increasing the likelihood of incidental vocabulary learning (Alibakhshi et al., 2025; Karimi and Nasouri, 2024; Lee, 2023; Wang and Feng, 2025; Wu et al., 2025).

Digital games can be considered one such environment; Digital games, with their engaging nature, interactive features (Oh et al., 2023; Quadir et al., 2022; Ryu, 2022), and collaborative learning experience (Prastiwi and Lestari, 2025), possess significant potential for vocabulary learning by facilitating both natural and implicit learning. They are a remarkable tool for vocabulary instruction because they combine entertainment and teaching processes, increasing participation and motivation in the classroom (Reinders, 2012). The most fundamental contribution of these games to vocabulary instruction is their long-term, repeated exposure to the language (Vnucko and Klimova, 2023). In other words, frequently exposing students to new words helps them remember their meanings, and vocabulary gradually accumulates as students play the games (Rasti-Behbahani, 2021). Learners in digital games not only learn new words but also remember and use them in real-life situations (Bakar and Nosratirad, 2013). Indeed, research shows that gamifying vocabulary teaching increases student engagement and supports long-term memory retention through repeated exposure and reinforcement (Chowdhury et al., 2024). Nation (2001) points out that, in the context of second-language learning, the higher the frequency of exposure, the higher the rate of vocabulary acquisition. From this perspective, the duration of exposure to the target language—namely, engagement in digital games—can be regarded as an important variable in the development of vocabulary knowledge.

Motivation is one of the psychological variables that can be affected by digital game-based learning (Ahmed et al., 2022). Learning is not limited to rigid rules and intensive memorisation; therefore, motivation is a crucial factor in guiding students to study and think (Prensky, 2007). Motivation is defined as what drives a person to make a specific commitment, participate in an activity, exert effort, and continue the action (Dörnyei and Ushioda, 2011). Motivation is an important and widespread behavioral determinant for students, teachers, and administrators (Pintrich and Schunk, 2013). According to research, human behavior is influenced by motivation in terms of the choice of a particular activity, commitment to that activity, and the effort invested in it (Dörnyei et al., 2006; Pang, 2021; Yang, 2021). Various studies have shown that playing games also increases motivation among learners (Boudadi and Gutiérrez-Colón, 2020; Saleem et al., 2022). However, since students, teachers, and researchers widely accept games as an effective way to increase interest and motivation, an increasing portion of research on vocabulary learning has focused on digital game-based vocabulary learning (Tsai and Tsai, 2018). Accordingly, it can be said that in the digital game-based vocabulary learning process, motivation plays a significant role, as well as the duration of exposure to the target language through playing digital games. Indeed, two students with the same amount of game time may have different levels of motivation, which can affect the vocabulary learning process. Over the last decade, researchers have found that technology can improve students' vocabulary development and reading comprehension skills (Ahmadi and Reza, 2018; Belo et al., 2016) and increase student motivation (Sabirli and Çoklar, 2020). Students may find it challenging to focus on explicit vocabulary learning for extended periods consciously; motivation plays a crucial role in vocabulary acquisition (Nation, 2001).

Anxiety is another affective factor influencing vocabulary learning. The literature indicates that this type of anxiety weakens learners' motivation to learn, increases their tendency to avoid communication in the target language, and negatively affects academic performance (Said and Weda, 2018). In the context of L2 learning, anxiety can affect language learners' performance (Liu, 2022; Teimouri et al., 2019). Indeed, it has been determined that foreign language anxiety affects academic achievement (Hashemi, 2011; Song, 2024), and students with high anxiety levels struggle with vocabulary learning (Kühl and Wohninsland, 2022). In this context, there has been an increased demand for options that can reduce anxiety levels in individuals during the language learning process. Digital games have emerged as one of the tools available for this process. Indeed, research has shown that vocabulary learning through digital games significantly reduces foreign language learning anxiety compared to traditional methods. Furthermore, it has been found that students with high anxiety levels develop complex cognitive and behavioral strategies in some digital game environments and achieve higher learning outcomes (Al-Aosail et al., 2024; Yang et al., 2024). Therefore, individuals' anxiety levels have been reduced, and their vocabulary learning performance has been increased through digital games. Students with high anxiety levels may avoid language learning (Khan and Zafar, 2010), find the input hard to understand (Chen, 2015), and have difficulty sticking to the vocabulary learning process (Bin-Hady, 2021). However, digital games can reverse this situation by offering an environment where mistakes are considered natural, and students can learn without fear of judgment (Vnucko et al., 2024). Students with reduced anxiety tend to develop their vocabulary more efficiently (Alemi et al., 2015). When the data in the literature are evaluated generally, it shows that vocabulary learning from digital games has the potential to reduce anxiety, and individuals with low anxiety exhibit higher learning performance. Therefore, it can be said that the relationship in question is reciprocal. However, in this study, anxiety is not conceptualized as a result of playing digital games; rather, it is treated as an individual learner characteristic that can affect incidental vocabulary learning. From this perspective, anxiety is considered an affective variable that can shape how learners process and benefit from the linguistic input they encounter during digital gaming. Therefore, examining anxiety as a predictor variable can provide important insights into how learners' affective characteristics influence incidental vocabulary acquisition in digital gaming environments. In particular, understanding the contributions of unstructured digital games, especially those used for instructional purposes, to incidental vocabulary learning in L2 requires knowledge of several variables. A review of the relevant literature reveals a generally negative relationship between individuals' motivation and anxiety levels (Ahmetovic et al., 2020; Basco and Han, 2016). Accordingly, it can be stated that higher levels of anxiety during the language learning process are associated with lower levels of learner motivation (Wu et al., 2022). In addition, anxiety has been found to be negatively related to learning performance. For this reason, many researchers have emphasized the importance of reducing anxiety through the use of effective instructional strategies (Pabro-Maquidato, 2021; Tee et al., 2020). By providing rich, context-based language input and an interactive learning structure, digital games create opportunities for meaningful and repeated exposure to target-language vocabulary, which may, in turn, positively influence learning performance as well as affective variables.

The existing literature has generally examined variables such as vocabulary learning, motivation, anxiety, and level of exposure within a relatively binary framework (Al-Aosail et al., 2024; Yang et al., 2024). Moreover, studies conducted within the context of digital games have predominantly focused on games developed for educational purposes, while the language-learning potential of commercially available games that learners choose to engage with voluntarily has received limited attention (Chen and Hsu, 2020; Peterson et al., 2020; Ranalli, 2008). In addition, empirical research addressing the incidental learning of foreign vocabulary through digital games among middle school students remains limited (Hung et al., 2018; Kazu and Kuvvetli, 2023). These studies have particularly examined the effects of digital games on motivation and anxiety in foreign language learning. However, data on how individuals with high foreign-language learning motivation or low anxiety learn vocabulary through digital games are pretty limited. In this context, determining how the process of playing games, foreign language learning motivation, and anxiety explain individuals' levels of incidental vocabulary learning is an important topic. This research aims to reveal the extent to which these variables explain success in learning vocabulary from digital games through a hierarchical regression model. The hierarchical structure used in the model does not merely represent the statistical ranking of variables; it also reflects a theoretical framework explaining incidental vocabulary learning in digital game environments. Within this framework, game playing time is treated as a behavioral variable representing individuals' level of exposure to the target language, forming the fundamental condition for incidental vocabulary learning. After controlling for this exposure-based variable, affective learner characteristics such as motivation and anxiety were included in the model in the second stage. Thus, it is possible to examine how the linguistic input encountered in the game environment is processed by learners and how it is shaped by individuals' psychological characteristics. Therefore, the hierarchical model allows for explaining incidental vocabulary learning first in the context of behavioral interaction established with the game environment, and then in the context of affective characteristics that facilitate or limit this process.

### 1.1 Current study

This study aims to examine the factors influencing incidental vocabulary learning through digital games. The study will investigate how behavioral and affective variables such as game-playing time, foreign language learning motivation, and foreign language learning anxiety affect the vocabulary acquired by learners through digital games. Hence, the following research questions guided the current study.

1. What is the difference in vocabulary test scores between learners who play digital games and those who do not?
2. How does digital game playing time predict incidental vocabulary learning?
3. How do foreign language learning motivation and anxiety affect incidental vocabulary learning?
4. How do game-playing time, foreign language learning motivation, and anxiety relate to each other in explaining incidental vocabulary learning?
5. How do game-playing time, motivation, and anxiety together explain incidental vocabulary learning?

## 2 Methods

### 2.1 Participants

The data for this study were collected from three public middle schools. The schools were included in the study after obtaining institutional permission processes and administrative approvals. The schools where data were collected were selected from among public middle schools where the necessary permissions could be obtained and the study could be implemented. The administrators of the selected schools were informed about the purpose and implementation process of the study. Students attending these schools who were identified as playing the digital game (League of Legends) considered in the study were then invited to participate in the data collection process. Students attending the same schools who stated that they did not play any digital games were also invited to participate in the study. Participants were classified as digital game players and non-digital game players based on self-reports obtained from the students. The group that played digital games consisted only of students who reported actively playing the digital game League of Legends. The group that did not play digital games consisted of students who stated that they did not play any digital games. Students who stated that they had previously played digital games but later stopped were not included in this group. This classification was applied to enable a clearer distinction between students exposed to English words through playing digital games and those without such exposure. Care was taken to ensure that the size of this group was close to that of the group that played digital games for the purpose of comparison. In addition, care was taken to ensure a balanced distribution of grade levels between the two groups. Participants were selected only from among students who volunteered to participate in the study. Descriptive information about the participants is shown in Table 1.

**Table 1:** Distribution of the study group by gender and grade level.

Variable	Category	Gamer	Non-gamer
Gender	Male	502	324
	Female	101	277
Grade	5th	129	140
	6th	130	164
	7th	187	147
	8th	157	150

The study group for the current research consisted of middle school learners, divided into two groups according to their digital game-playing habits. These learners were classified as the digital game-playing group (603) and the non-digital game-playing group (601). Of the learners in the digital game-playing group, 502 were male, and 101 were female. When the distribution of this group by grade level was examined, there were 129 learners in 5th grade, 130 in 6th grade, 187 in 7th grade, and 157 in 8th grade. In the non-digital game-playing group, there were 324 male and 277 female learners. The grade-level distribution of this group was 140 learners in 5th grade, 164 in 6th grade, 147 in 7th grade, and 150 in 8th grade. Additionally, in this study, we did not use a standardized assessment tool that directly measures participants' initial English proficiency levels. However, we took certain measures to minimize potential proficiency differences among participants. Specifically, we selected all students from the same schools and grade levels and identified them from among students following the same national English teaching program. This approach was intended to ensure that participants shared a largely similar educational environment in terms of exposure to formal English instruction and educational background. Nevertheless, this situation is reported as one of the limitations of the study in the limitations and recommendations section. To demonstrate that vocabulary scores were primarily game-related, only the vocabulary test scores of the non-digital game-playing group were used; analyses of the digital game-playing group were performed using data from other tests.

### 2.2 Measures

#### 2.2.1 Frontiers vocabulary knowledge assessment form

We used the Vocabulary Knowledge Assessment Form developed by Simşek and Direkci (2019) to assess learners' vocabulary levels acquired through digital game play. This form was developed using English words from the digital game League of Legends, identified through observations of the game environment and online broadcasts. During the form's development process, League of Legends was among the most-played digital games in Türkiye, and the specific English terms (dodge, flash, ignite, insect, smurf, spell, ult, etc.) are frequently used in gameplay. The vocabulary items included in the test have been selected from expressions commonly encountered in the League of Legends gaming environment, but they are not limited to game-specific commands or technical jargon. On the contrary, these items represent unique English words that are also used in general language. Words such as flash, spell, and ignite are commonly used in English and require understanding of their meaning within the context of the game. From this perspective, the gaming environment serves as a natural exposure context where students repeatedly encounter these words. Therefore, the assessment tool is designed to measure vocabulary knowledge that can develop through contextual exposure and implicit learning during gameplay, rather than the memorization of game commands. Since the test was developed in Türkiye and focuses on Turkish learners, it is well-suited for this study. The data indicate that League of Legends is among the most-played digital games in Türkiye, supporting the objectives of the present research. During the test development process, the relevant literature was reviewed, expert opinions were consulted, and a pilot was conducted. The reliability of the measurement tool, which includes 82 English words identified as frequently used within the context of the game, was examined using the KR-20 coefficient, and the test was found to have a high level of reliability (KR-20 = 0.98). In the present study, the scores obtained from this test were used as an indicator of learners' levels of incidental vocabulary acquisition from digital games.

#### 2.2.2 Foreign language learning motivation scale

Learners' Motivation to learn a foreign language was tested with the Motivation Scale for Foreign Languages, adapted into Turkish by Babacan and Acat (2022). The scale is based on the “Motivation for English as a Global Language Questionnaire” developed by Weger (2008) and adapted into Turkish as a valid and reliable measurement tool. The scale consists of 30 items. The construct validity of the scale was assessed using confirmatory factor analysis; the fit indices were acceptable, indicating good fit. The Cronbach's alpha for internal consistency of the scale was 0.94, indicating high reliability. Confirmatory factor analysis (CFA) was conducted to examine whether the factor structure of the scale was supported in the current data set. The analysis results showed that the factor structure of the scale reported in the original study was preserved in the current sample. In this study, the reliability coefficient was 0.85. In this study, total scores from the scale were used to indicate learners' motivation to learn a foreign language. It should be noted that the scale measures learners' general motivation toward foreign language learning rather than motivation specifically related to digital game contexts. In the present study, this construct was considered as an overall motivational disposition toward learning English. This broader motivational orientation was assumed to influence learners' engagement with English vocabulary encountered in digital game environments.

#### 2.2.3 Foreign language learning anxiety scale

The Foreign Language Learning Anxiety Scale, developed by Baş (2013), was used to assess learners' anxiety regarding foreign language learning. The scale, designed to measure anxiety among secondary school learners during foreign language learning, comprises 27 items. The construct validity of the scale was tested using exploratory factor analysis; the KMO value was 0.932, and Bartlett's sphericity test was significant. The reliability of the scale was determined using Cronbach's alpha. The total reliability coefficient was reported as 0.93. Confirmatory factor analysis (CFA) was conducted to examine whether the factor structure of the scale was supported in the current data set. The analysis confirmed that the factor structure reported in the adaptation study was also preserved in the current sample. The reliability coefficient was calculated as 0.86, and the total scores obtained from the scale were used to indicate learners' foreign language learning anxiety levels. It should be noted that the scale measures learners' general foreign language learning anxiety rather than anxiety specifically associated with digital game contexts. In the present study, this construct was conceptualized as learners' overall foreign language learning anxiety toward English. This broader anxiety orientation may influence how learners attend to, engage with, and process English vocabulary encountered in digital game environments.

### 2.3 Procedure

Before the study began, we obtained ethical committee approval, Ministry of National Education permission for implementation, and necessary data-collection permissions from the participating schools. We collected data from middle school learners in the school environment during class hours under the supervision of teachers and researchers. We identified those who played the digital game League of Legends. Next, to allow for comparison in the context of vocabulary testing, we identified learners who did not play any digital games and who volunteered to participate in the study. We administered the vocabulary test to both groups. Additionally, we asked learners who played digital games to report the time they spent playing. We asked participants to estimate approximately how much time they dedicate to playing the digital game (League of Legends) on a weekly basis. This information was collected based on self-reports, where students indicated how many hours they typically spend playing digital games within a week. Finally, to assess learners' motivation and anxiety related to foreign language learning while playing digital games, we administered the Foreign Language Motivation Scale and the Foreign Language Learning Anxiety Scale, respectively. The achievement tests and scales were administered across grade levels, and care was taken to ensure a balanced distribution of grades. All measurement tools were administered in a single data collection session conducted within the classroom setting. The data collection process was completed within two class periods. During the first class period, we provided the students with information about the purpose and content of the study and ensured they completed the vocabulary test. In the second class period, we collected data using motivation and anxiety scales. The entire data collection process took approximately 50–60 min. The entire procedure was conducted under the supervision of the researchers. After completing data collection, we coded the data and prepared it for statistical analysis. We removed incomplete or incorrectly completed forms from the dataset and conducted analyses on the remaining valid forms.

### 2.4 Data analysis

In the data analysis, we first confirmed that the data were normally distributed. Then, we calculated descriptive statistics for the variables considered in the study. Subsequently, we conducted an independent-samples *t*-test to compare vocabulary scores between the two groups in the study: those who played digital games and those who did not. Prior to conducting the independent samples *t*-test, the assumption of homogeneity of variance was examined using Levene's test. In addition, Cohen's d was calculated to estimate the effect size of the difference between the groups. To determine the direction and strength of the linear relationships between the variables, we used Pearson product-moment correlation analysis. We conducted this analysis to identify relationships among variables and to assess the presence of multicollinearity before proceeding with hierarchical regression. The primary purpose of using hierarchical multiple regression in this study is to identify variables that predict incidental vocabulary learning from digital games. In addition, this analysis allows the inclusion of predictor variables in a model-theoretic order and the stepwise examination of the additional explanatory contribution of each group of variables to the dependent variable. Assuming that exposure to these games for a specific period is necessary for vocabulary learning from digital games to occur, we treated this variable as the primary predictor. In the second model, the motivation and anxiety variables, which we presented together, were included in the same block because they are affective variables addressed within the same theoretical framework. This approach aims to evaluate the additional contribution that affective factors make to word learning, after controlling for playing time. Thus, the explanatory power of affective characteristics on word learning could be examined when the effect of a behavioral variable was kept constant. In the hierarchical regression analysis, we calculated the *R*^2^, Δ*R*^2^, and F change values for each model. We evaluated the relative effects of the predictor variables on the dependent variable through standardized beta coefficients (β). The assumptions of the regression analysis were checked; multicollinearity was examined using tolerance and VIF values and was found to be at acceptable levels for all variables.

## 3 Results

In this study, we examined the variables predicting incidental vocabulary learning from digital games. To determine whether the vocabulary acquired by learners who played digital games was game-related, we first compared their vocabulary knowledge with that of a group who did not play any digital games. We then conducted analyses using data from a group of 603 learners who played games. Descriptive statistics for the variables included in the study are presented in Table 2.

**Table 2:** Descriptive statistics for variables.

Variable	*N*	Mean (M)	Standard deviation (SD)
Word test score (non-gamers)	601	2.99	2.89
Word test score (gamers)	603	40.60	23.02
Weekly gaming time (league of legends)	603	10.19	5.90
Foreign language learning motivation	603	115.54	18.08
Foreign language learning anxiety	603	88.70	12.64

The mean score on the vocabulary test used in the current study for learners who do not play digital games was 2.99 (SD = 2.89), whereas for learners who play digital games it was 40.60 (SD = 23.02). We found a statistically significant difference between the two groups (*t* = 39.724, *p* < 0.05). The effect size was extremely large (Cohen's *d* = 2.29). Therefore, learners who play digital games scored significantly higher in recognizing lexical items that frequently occur in the game environment. Additionally, the average weekly gaming time of the learners participating in the study was 10.19 h (SD = 5.90); the average foreign language learning motivation score was 115.54 (SD = 18.08); and the average foreign language learning anxiety score was 88.70 (SD = 12.64). Pearson correlation analysis was performed to examine the relationships between these variables, and the results are shown in Table 3.

**Table 3:** Pearson correlation coefficients between variables.

Variables	1	2	3	4
1. Word test score	—			
2. Weekly gaming time	0.463^*^	—		
3. Foreign language learning motivation	0.310^*^	0.107^*^	—	
4. Foreign language learning anxiety	−0.269^*^	−0.163^*^	−0.322^*^	—

^*^Correlation is significant at the 0.05 level.

According to Table 2, there is a moderately positive and significant correlation between vocabulary test scores and weekly game playing time (*r* = 0.463, *p* < 0.05). While there is a low-to-moderate positive correlation between vocabulary test scores and motivation for learning a foreign language (*r* = 0.310, *p* < 0.05), a low-level negative correlation was found between anxiety about learning a foreign language and vocabulary test scores (*r* = −0.269, *p* < 0.05). When the relationships between the independent variables are considered, a moderately negative correlation is found between motivation and anxiety (*r* = −0.322, *p* < 0.05), and the relationships between weekly game playing time and motivation and anxiety are at a low level. Based on these findings, there is no significant multicollinearity prior to the regression analyses. We conducted a two-stage hierarchical regression analysis to determine the variables predicting incidental vocabulary learning from digital games. The results of the analysis are presented in Table 4.

**Table 4:** Results of hierarchical regression analysis.

Variable	*B*	SH	β	*t*	*p*
Model 1	22.20	1.66		13.37	<0.05
Weekly gaming time	1.81	0.14	0.463	12.80	<0.05
Model 2	11.67	9.31		1.25	0.210
Weekly gaming time	1.63	0.14	0.418	12.02	<0.05
Foreign language learning motivation	0.29	0.05	0.224	6.19	<0.05
Foreign language learning anxiety	−0.23	0.07	−0.128	−3.51	<0.05

In the first model, within the hierarchical regression framework, we included only weekly gaming time. We found a statistically significant effect [*F*_(1,601)_ = 163.84, *p* < 0.05]. Weekly gaming time positively and significantly predicted vocabulary test scores (β = 0.463, *p* < 0.05) and accounted for 21.4% of the total variance in the dependent variable (*R*^2^ = 0.214).

In the second model, in addition to weekly gaming time, we included variables for foreign-language learning motivation and foreign-language learning anxiety. The analysis revealed that this model was statistically significant [*F*_(3,599)_ = 84.56, *p* < 0.05]. The total variance explained by the second model increased to 29.8%. A significant increase in the explained variance occurred with the addition of motivation and anxiety variables to the model (Δ*R*^2^ = 0.083, *p* < 0.05). In the final model, weekly playtime showed the largest standardized effect among the predictors included in the model (β = 0.418, *p* < 0.05). Motivation for learning a foreign language positively and significantly predicted vocabulary test scores (β = 0.224, *p* < 0.05). In contrast, anxiety about learning a foreign language negatively and significantly predicted vocabulary test scores (β = −0.128, *p* < 0.05).

## 4 Discussion

In the present study, we examined the key variables predicting students' incidental vocabulary acquisition within a defined digital game framework. The analysis results indicate that digital games provide a learning environment that may be related to incidental word acquisition in second language learning. The significant achievement difference observed between students who played digital games and those who did not indicated that students who played without a conscious instructional plan acquired game-related vocabulary incidentally. However, it should also be acknowledged that the learning processes occurring in digital game environments may not be entirely incidental. For purposes such as improving game performance, students may consciously focus on unfamiliar words encountered during gameplay, look up their meanings, or use various learning strategies to understand game-related terminology. Therefore, vocabulary learning in digital game contexts may involve a dynamic interaction between incidental exposure and intentional learning processes. Overall, the study results are consistent with studies showing that digital games support incidental vocabulary acquisition in second language learning (Calvo-Ferrer and Belda-Medina, 2021; Lee, 2023; Weisi and Hajizadeh, 2025). It should be noted that the vocabulary test used in this study consisted of lexical items frequently encountered in the digital game environment. Therefore, the observed difference between the groups should primarily be interpreted as a difference in the recognition of game-related vocabulary rather than as an indicator of general L2 vocabulary proficiency. An important factor to consider when interpreting the findings is the gender imbalance between the groups. While the group that played digital games consisted predominantly of boys, the group that did not play games was more balanced. Previous research on out-of-school English suggests that male students participate more frequently in digital gaming activities, which may lead to greater exposure to English outside the classroom (Sylvén and Sundqvist, 2012; Sundqvist and Sylvén, 2016). Therefore, some of the observed differences in vocabulary may also be related to gender-related differences in gaming behavior and language exposure.

Studies on game-based learning and digital games have shown that these games create a pressure-free learning environment for students (Qasim, 2021). This situation may have supported the incidental learning process. The frequent contextual repetition of words learned in these environments may also naturally and incidentally support word learning (Chowdhury et al., 2024; Lai and Chen, 2025). Although they have different characteristics, in general, features such as the use of words in different contexts in digital games and the frequent repetition of visual, auditory, and interactive inputs help learners make healthy form-meaning associations with words. In this context, both the depth and retention of incidental word learning increase (Chen and Hsu, 2020; Loor and Vivero, 2025; Shahiwala and Rahul, 2025; Rasti-Behbahani and Shahbazi, 2022; Vnucko and Klimova, 2023). The results of the current study suggest that digital games may contribute to increased exposure to language input by offering a rich learning environment that supports incidental vocabulary learning and allows them to reconstruct this input through meaningful interactions. Indeed, previous studies have supported this finding, showing that exposure to linguistic input is a fundamental prerequisite for incidental vocabulary learning (Eckerth and Tavakoli, 2012; Rasti, 2020). Although exposure frequency is vital for incidental language learning, this process cannot be explained solely by the frequency of learners' exposure to words. From a cognitive perspective, repeated encounters with words in game environments with multimedia features can activate various learning mechanisms. First, repeated encounters can strengthen encoding processes by allowing for the simultaneous processing of visual, auditory, and contextual cues (Chun and Jiang, 1998; Mayer, 2005; Paivio, 1986; Teng, 2023). Second, repeated encounters can support retrieval processes, helping students recall words they have previously encountered during gameplay (Karpicke and Roediger, 2008; Nation, 2001; Roediger and Karpicke, 2006; Webb, 2007). Third, interactive tasks in games can promote deeper levels of processing, increasing the retention of vocabulary knowledge (Craik and Lockhart, 1972; Hulstijn and Laufer, 2001; Keating, 2008; Laufer and Hulstijn, 2001). Moreover, the learning potential of digital games should not be explained solely by exposure frequency. Game environments incorporate multidimensional learning features such as contextual salience, emotional engagement, goal-directed interaction, and problem-solving activities (Gee, 2003; Peterson, 2016; Reinders and Wattana, 2015). These features can make words more attention-grabbing and meaningful for students. Digital game-based learning environments can enhance vocabulary learning by supporting students' positive emotional experiences and processing words in meaningful contexts (Vnucko and Klimova, 2023; Zou et al., 2021). The narrative structures of games, competitive elements, and collaborative tasks can encourage students to pay more attention to language forms, helping them engage in deeper cognitive interaction with vocabulary input (Yang and Quadir, 2018). Therefore, the effectiveness of vocabulary learning in digital games may depend not only on the frequency of repetition but also on the interaction of cognitive, contextual, and affective factors within the game (Dixon et al., 2022).

Weekly gaming time was one of the variables we examined in relation to students' vocabulary achievement. The analysis results showed a moderately positive correlation between weekly gaming time and incidental vocabulary acquisition from digital games. Hierarchical regression analysis based on this data revealed that gaming time was the strongest predictor of incidental vocabulary acquisition among the variables considered in this study. Therefore, although exposure frequency appears to be an important factor associated with incidental vocabulary learning, it should be acknowledged that other unmeasured variables may also contribute to this process. This result is consistent with related studies (Nation, 2022; Schwab and Lew-Williams, 2016; Teng, 2016). One study examining the effects of exposure frequency is Shin and Kim (2023). This study showed that repeating target words a different number of times, in other words, increasing the frequency of exposure to target words, positively and significantly increased vocabulary acquisition. Another study conducted within this framework examined incidental word learning and found that repeated exposure produces lasting effects, both immediately and in the long term, on vocabulary dimensions such as word recognition and meaning association (Nie et al., 2022). When we evaluate the results of the current study and the literature together, we can say that the frequency of exposure to digital games is one of the main determinants of incidental word learning. Although gaming time showed the largest standardized coefficient in the regression model, the explained variance indicates that a substantial proportion of vocabulary learning may also be influenced by other cognitive, contextual, and instructional factors not examined in the present study.

Another variable that significantly and positively influences incidental vocabulary learning and is considered in this study is motivation. Analyses conducted within the research framework show that students with high motivation to learn a foreign language achieve higher scores in in-game incidental vocabulary learning. In this context, the results emphasize that affective variables, as well as gameplay time, can play a decisive role in vocabulary learning. Therefore, in line with Schmidt's (2001) awareness hypothesis, motivation facilitates deeper processing of vocabulary learning at the cognitive level. In this context, it can be said that motivation has a holistic effect, positively influencing learners' immediate attention processes and structuring their self-perceptions toward L2. When we evaluate the results within the framework of Dörnyei's (2009) Second Language Motivational Self-System, we can say that digital games offer powerful, meaningful learning contexts that can nurture learners' ideal second-language selves. More specifically, the immersive and goal-oriented nature of digital games may allow learners to imagine themselves as competent users of the target language within the game environment, thereby strengthening their Ideal L2 Self. At the same time, elements such as team collaboration, task completion, and achievement systems may create expectations related to performance and responsibility, which can be associated with the Ought-to L2 Self. The motivation scale used in this study measures general foreign language learning motivation rather than motivation specific to digital games. However, this broader motivational orientation may influence how students interact with English words encountered while playing games. Students with high general second language (L2) learning motivation may be more attentive to linguistic input and more willing to process new or unfamiliar words encountered in digital game environments. In this context, the autonomy-supportive structure of game environments, opportunities for instant feedback, and goal-oriented progress mechanisms contribute to the sustainability of the learning process by strengthening intrinsic motivation (Al-Aosail et al., 2024; Shahiwala and Rahul, 2025). Within this scope, it can be said that there is a reciprocal relationship between vocabulary learning through digital games and motivation for learning a foreign language. While digital game environments have the potential to increase learning motivation, this motivation can also strengthen incidental vocabulary learning. Another finding of the current study is that anxiety about learning a foreign language negatively predicts vocabulary learning. The findings reveal that anxiety has a limiting effect on cognitive processes. The decrease in learning performance parallel to the increase in anxiety level can be explained by the shift of cognitive resources away from task-related processes toward anxiety-focused thoughts (Chen and Chang, 2009; Gullo et al., 2025). However, the limited predictive power of anxiety suggests that digital game-based learning environments offer a lower threat perception compared to traditional classroom contexts. Learning environments that tolerate mistakes, opportunities for anonymous interaction, and individual learning pace can partially offset the destructive effects of anxiety on the learning process by reducing the fear of negative evaluation (Al-Aosail et al., 2024; Tai and Choi, 2025; Yang et al., 2024). In this context, digital games can be considered alternative learning contexts that offer safe spaces for experimentation and exploration for students with high anxiety levels (Hwang et al., 2017). The anxiety scale used in the current study measures general foreign language learning anxiety rather than contextual anxiety specific to digital games; however, this broader anxiety tendency may still influence how students respond to the linguistic input they encounter while playing games. Students with higher levels of foreign language anxiety may experience greater difficulty processing new or unfamiliar words encountered in digital game environments. Therefore, considering the relevant literature and the results of the current study together, it can be said that digital games may be associated with lower levels of foreign language learning anxiety, and students with lower levels of anxiety tend to demonstrate higher vocabulary scores.

Overall, this study revealed that incidental vocabulary learning through digital games has a multidimensional and dynamic structure. Playing time emerged as a determining variable in incidental vocabulary learning. However, we also found that affective factors, such as foreign-language learning motivation and anxiety, influence learners' processing of linguistic input encountered in game environments. Therefore, the data show that incidental vocabulary learning is shaped not only by encounter frequency but also by the learner's existing affective characteristics. These results highlight the need to consider behavioral indicators alongside affective variables. In this respect, the present study makes a significant contribution to the existing literature by addressing the linguistic input potential offered by digital games in out-of-classroom learning contexts not only quantitatively but also within a learner-centered and affective process-integrated framework (Calafato and Clausen, 2025; Leona et al., 2020; Rezai and Goodarzi, 2025; Uztosun and Sundqvist, 2025; Vnucko et al., 2024; Zhang and Lai, 2024). Theoretically, these findings contribute to the literature by showing that incidental vocabulary acquisition in digital game environments results from the interaction between exposure frequency, affective variables, and cognitive processing mechanisms. These results suggest that incidental vocabulary learning should not be conceptualized as merely a passive exposure process, but rather as one shaped by students' levels of engagement, motivational orientations, and affective states. From a practical perspective, the findings indicate that digital games can be strategically integrated as a complementary teaching tool in language learning environments. Game environments that provide rich contextual language input, offer repeated exposure opportunities, and involve meaningful interactions can particularly support vocabulary development in out-of-class learning contexts. Therefore, teachers and curriculum developers may consider integrating game-based activities (Hamari et al., 2014; Plass et al., 2015) and digital game environments that support vocabulary learning (Sundqvist and Wikström, 2015; Peterson, 2016) into language teaching processes. Additionally, it should be noted that the present study employed a cross-sectional correlational design. Therefore, the findings should be interpreted as associational rather than causal relationships.

## 5 Conclusion

The findings obtained from the two groups, those who play digital games and those who do not, in the current study suggest that playing digital games may be associated with incidental vocabulary learning. First, we determined a significant positive correlation between learners' vocabulary test scores and their game-playing time. In this regard, it can be argued that digital games not developed within a specific teaching plan may still be associated with vocabulary learning in a second language (L2) context. However, it should be remembered that the concept of “digital game” is comprehensive; there are many different types of digital games with distinct features, and these games offer different contexts, so different results may emerge in studies that consider different digital games. Another variable that may be effective in incidental vocabulary learning from digital games is motivation. As a result of our analysis, we determined that L2 learning motivation significantly and positively predicted learners' vocabulary test scores. In this context, we can say that individuals' L2 learning motivation can be effective in incidental vocabulary learning through digital games. Another variable we examined was L2 learning anxiety. The findings showed that anxiety can negatively affect the incidental vocabulary learning process, playing an inhibitory role. Therefore, this study can be said to contribute to our understanding of incidental vocabulary acquisition by integrating both behavioral (exposure) and affective (motivation, anxiety) variables. By considering English exposure in the context of digital gameplay alongside students' motivation and anxiety levels, we gain a more comprehensive understanding of how these factors are related to vocabulary learning. The integration of these variables demonstrates that not only the amount of exposure but also the emotional responses learners have to the learning context play a significant role in incidental vocabulary acquisition. This theoretical contribution enhances our understanding of the relationship between external language exposure and internal emotional states in the process of vocabulary development. Overall, evaluating the results of the current research, we can say that the most significant influence on incidental vocabulary learning from digital games is the duration of play. In addition, we found that affective factors, such as foreign-language learning motivation and anxiety, also significantly affect incidental vocabulary learning. The results of this study highlight the need to consider students' affective states when developing digital game-based learning strategies, providing an important reference for future educational practices.

## 6 Limitations and future research

This study has some limitations. First, this research is limited to data collected only from middle school learners. Therefore, it is recommended that future studies collect data from multiple groups to generalize the results or identify differences between age groups. In particular, future research should investigate whether the relationships observed in this study hold similarly across different developmental stages, such as adolescents, university students, or adult learners. Differences in cognitive maturity, language learning experience, and forms of participation in digital games may lead to varying learning outcomes across age groups. Similarly, this research was conducted by collecting data from a few schools in a specific geographic region and focused on Turkish-speaking learners' learning of English vocabulary as an L2. It is suggested that similar research be conducted in other languages and regions. Therefore, the findings should be interpreted within this specific cultural and educational context. Cultural factors, educational practices, and the level of students' exposure to English outside of school may vary significantly across countries and learning environments. Future studies conducted in different cultural contexts and educational systems will contribute to the evaluation of the cross-cultural validity of these research findings. This study specifically focuses on vocabulary exposure occurring through digital gameplay. Other potential sources of English exposure outside of digital games (such as English classes, social media use, online video content, or other extracurricular activities) were not directly measured in the present study. Additionally, participants' initial English proficiency was not assessed. Another limitation of the study is the measurement tools. The results were obtained from the measurement tools used in the study; therefore, different measurement tools may yield different results. The vocabulary test is a measurement tool explicitly prepared for a digital game. Different results may be obtained across different digital games. Additionally, the vocabulary items included in the test were derived from a specific commercial digital game (League of Legends). Therefore, the findings obtained may reflect not only the general impact of digital games but also the linguistic features and interaction patterns specific to this game. In this context, it should also be considered that the observed difference between students who play digital games and those who do not may be due not only to the effectiveness of learning but also to the greater exposure of players to these words within the game environment. The random language-learning scenarios of different digital games can also be examined with respect to data diversity. Additionally, it should be noted that in this study, the weekly gaming time was measured based on participants' self-reported estimates. Self-reported measurements of time use may be subject to recall bias and may not fully reflect the actual amount of time spent playing digital games. Future research could benefit from utilizing objective tracking data or digital game logs to obtain more accurate measurements of gaming duration. In addition, the scales used in this study address affective factors such as motivation and anxiety. However, additional affective factors that may influence learners' vocabulary learning processes warrant attention in future research. Furthermore, another limitation of this study concerns the measurement of motivation and anxiety. The instruments used in this research measure general foreign language learning motivation and anxiety, rather than motivation and anxiety specific to digital game environments. Future research could benefit from utilizing context-specific measurements to examine how affective factors related to gaming influence vocabulary learning processes. Another limitation of the study concerns potential confounding variables. The digital game-playing group was predominantly male, whereas the non-playing group showed a more balanced gender distribution. Gender and grade level were not included as control variables in the regression analyses and may have influenced vocabulary outcomes. Finally, in future research, the effect of digital games on vocabulary learning can be examined in greater depth through longitudinal designs. This method can also be used to investigate the impact of digital games on different language skills.

## Data Availability

The data presented in this study are available on reasonable request from the corresponding author.
